# Amalgam tattoo: a cause of sinusitis?

**DOI:** 10.1590/S1678-77572010000100016

**Published:** 2010

**Authors:** José Luiz Santos PARIZI, Gisele Alborghetti NAI

**Affiliations:** 1 DDS, Professor, Department of Pathology, University of Western São Paulo, Presidente Prudente, SP, Brazil.; 2 MD, PhD, Professor, Department of Pathology, University of Western São Paulo, Presidente Prudente, SP, Brazil.

**Keywords:** Amalgam, Tattoo, Dental restorative material, Sinusitis, HLA-DR

## Abstract

Little attention has been paid to the toxicity of silver amalgam fillings, which have been used over the centuries in Dentistry. Amalgam particles may accidentally and/or traumatically be embedded into the submucosal tissue during placement of a restoration and perpetuate in such area. This article presents a case of amalgam tattoo and investigates whether it is related to the patient's repeated episodes of sinusitis. The patient was a 46-year-old woman with a 2 mm diameter radiopaque lesion in the right oral mucosa detected on a panoramic radiograph and presented as a black macula clinically. A complete surgical resection was carried out. The histopathological examination revealed deposits of dark-brownish pigments lining the submucosal tissue with adjacent lymphocytic inflammatory infiltrate and multinucleated giant cells phagocyting pigments. There was a negative staining for both iron and melanin. One year after lesion removal, the patient reported that the sinusitis crises had ceased after repeated episodes for years. It may be speculated that the inflammatory process related to amalgam tattoo seems to lead to a local immune response that causes sinusitis because it enhances the human leukocyte antigen DR (HLA-DR) tissue expression.

## INTRODUCTION

Amalgam pigmentation, generally called amalgam tattoo, is a relatively common finding in the oral mucosa^1,8,13^. Gingiva and alveolar mucosa are the most common locations, and the mandibular region is more frequently affected than the maxillary region^1,10,13^.

Tissue reaction to amalgam can vary considerably and arise as a macrophage or chronic inflammatory response, usually in the form of foreign body reaction, or even not develop any response^1^.

This article presents a case of amalgam tattoo and investigates whether it is related to the patient's repeated episodes of sinusitis.

## CASE REPORT

A 46-year-old female patient presented with a 2 mm black macule on the oral mucosa of the posterior fornix on the right. The patient reported that a tooth with an amalgam filling had been extracted from that region 15 years before. Differential clinical diagnosis suggested were melanoma, melanocytic nevus, hematoma, hemangioma and amalgam tattoo.

**Figure 1:**
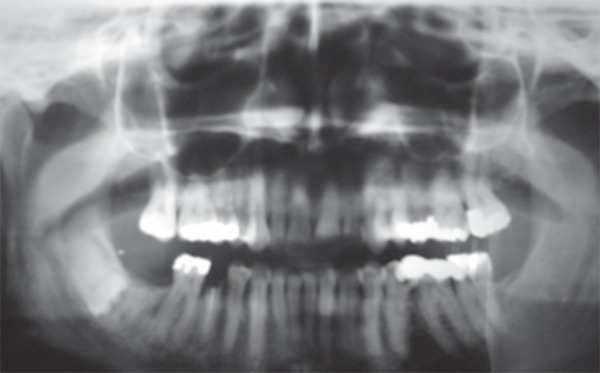
Panoramic radiograph showing a radiopaque lesion in the right oral mucosa (arrow)

A panoramic radiograph revealed a 2 mm radiopaque lesion on the right oral mucosa (Figure 1) in the same region as the clinical macula locus, and was consistent with a diagnosis of amalgam tattoo.

**Figure 2:**
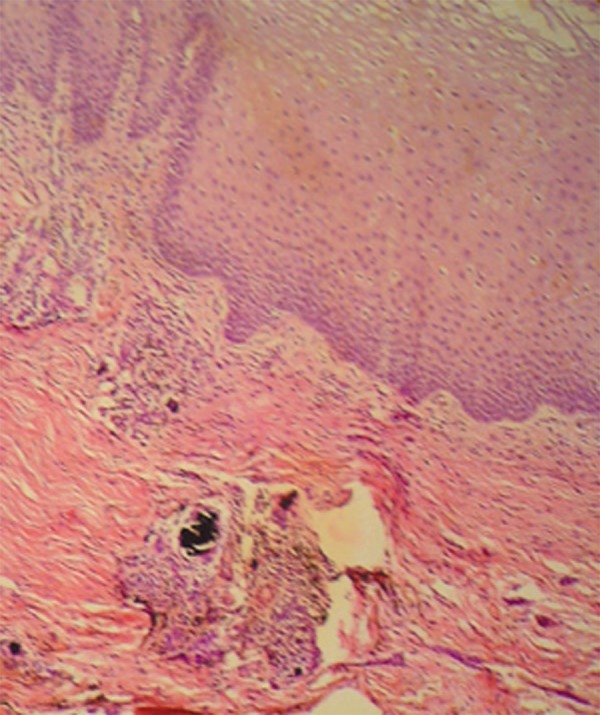
Epithelial acanthosis and brownish-black pigment deposits in the submucosal region (arrow) (Hematoxylin and eosin, ×100)

**Figure 3:**
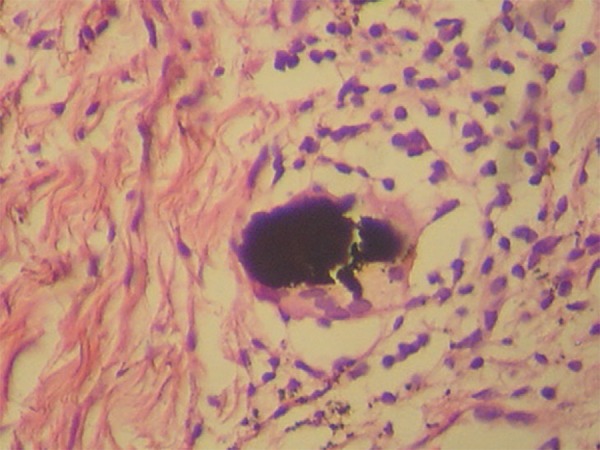
Multinucleated giant cells phagocyting brownish-black pigments (Hematoxylin and eosin, ×400)

**Figure 4:**
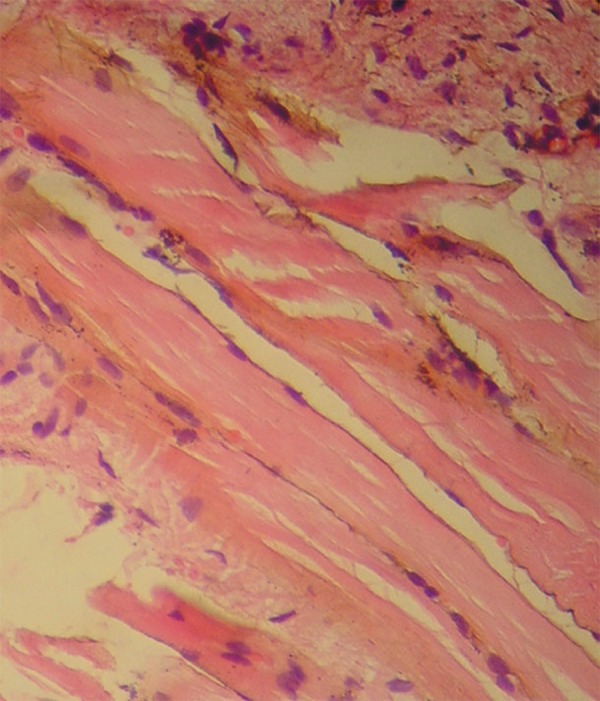
Impregnation of muscle cells by brownish-black pigments (Hematoxylin and eosin, ×400)

A complete surgical excision was carried out. Histopathological examination revealed epithelial acanthosis and brownish-black pigment deposits in the submucosa surrounded by a lymphocyte inflammatory infiltrate (Figure 2), and multinucleated giant cells phagocyting these pigments (Figure 3). Muscle cells were impregnated in the deeper layers (Figure 4). There was no iron or melanin staining. Final diagnosis for this patient was amalgam tattoo.

The patient had been suffering from repeated episodes of sinusitis for years, which had been diagnosed and treated by a physician. After excision of the lesion, the attacks ceased without any specific treatment. One year after surgery, no recurrence of sinusitis was observed.

## DISCUSSION

Amalgam tattoo is characterized by the deposit of restorative debris material composed of a mixture of silver, mercury, zinc, tin and copper in subepithelial connective tissue^8^ and is a common finding in the dental practice^1,8,13^. Clinically, it presents as an asymptomatic, ill-defined black macula or plaque whose main location is along the gingival margin and the alveolar mucosa, as in the present case^8^. Amalgam may accidentally and/or traumatically embed into the submucosa during a fitting of prosthetic crowns, removal of old amalgam fillings, extraction of restored teeth, endodontic treatment, and flossing shortly after a restorative dentistry procedure^1,8^. Also, amalgam might release some level of metals due to the galvanism between different alloys in the mouth (e.g.: a tooth restored with gold), and mercury may diffuse through soft tissue developing the amalgam tattoo^10^.

Connective tissue response to amalgam varies greatly and depends on the particle size and composition^2,4,8^. Buchner, et al.^1^ (2004) observed no reaction in 45% of the cases of amalgam tattoo, while 17% have a macrophage reaction, and in 38% a foreign body chronic inflammation was noted. In the present case, we observed the presence of a foreign body type chronic inflammation with pigment phagocytosis by multinucleated giant cells. While copper and zinc are rapidly lost from the region of the tattoo, mercury and tin are lost more slowly and, finally, only silver remains permanently in the tissues^7^.

Forsell, et al.^4^ (1998) reported that the inflammatory reaction to amalgam tattoo becomes more severe as mercury content in the tissue increases, and the delayed reaction has also been associated with the presence of silver. Probably, the immune response of the amalgam tattoo related-cases is the type IV hypersensitivity (delayed or cell-mediated hypersensitivity particularly with respect to CD4^+^ T-lymphocytes), which is similar to the contact allergy, and seems to be related to mercury in most cases^5^.

Nadarajah, et al.^12^ (1996) found an increased expression of class II major histocompatibility complex (MHC) molecules in monocytes, resident macrophages and mononuclear cell related to the mercury accumulation, which, thereby, promotes a persistent chronic inflammation and shifts in mononuclear cell subpopulations on the tattoo amalgam zone.

Several studies have shown that amalgam tattoos require no treatment intervention as they are a safe and innocuous restorative material and have not been reported to cause systemic health effects^1,15^. Amalgam tattoo should only be removed if no radiopaque particles are revealed radiographically because, in these cases, all other causes of oral pigmented lesions, especially melanomas, should be ruled out. However, Leite, et al.^8^ (2004) showed an increase in the level of human leukocyte antigen DR (HLA-DR) expression and metallothioneins (associated with heavy metal detoxification) in inflammatory cells, blood vessel walls and connective tissue fibers impregnated with amalgam debris. These findings demonstrate that residual elements of amalgam tattoo still have noxious local effects over tissues, and systemic effects could not be neglected.

Weaver, et al.^16^ (1987) reported a case of a patient who had had an amalgam tattoo for 2 years and complained of swelling and local soreness, sinusitis, headache, fatigue, and weight loss. Following complete removal of lesion, clinical symptoms ceased drastically, which may be suggestive of a direct relation to the amalgam tattoo. The patient of this case had a difficult clinical management of sinusitis for years that disappeared after removing the amalgam tattoo. We believe that the relationship between sinusitis and amalgam tattoo is that amalgam tattoo cause an increase in tissue-specific expression of the HLA-DR. Also, systemically free mercury in the amalgam may give rise to a type IV hypersensitivity reaction^11^, which can contribute to sinusitis attack.

Class II MHC antigens are coded for in a region called HLA-D, which has three sub-regions: HLA-DR, HLA-DP and HLA-DQ. In general, class II molecules present exogenous antigens to CD4^+^ T lymphocytes (helper T cells) expressed on macrophages, dendritic cells, B cells as well as in endothelial and fibroblast cell lines^6^. MHC molecules play a central role in regulating T cell-mediated immune response in two forms: 1. Since different antigenic peptides are bind to different class II gene products, an individual will mount a vigorous immune response against an antigen only if this individual inherits the gene(s) for those class II molecule(s) that can bind the nominal antigen and present it to helper T cells^6^. This could be the cause concerning the relation of different tissue reactions to the amalgam tattoo, justifying the absence of a reaction. 2. During intrathymic differentiation, only the T cells that can recognize self-MHC molecules are selected for export to the periphery. Thus, the type of MHC molecules that T cells encounter during their differentiation influences the functional capacity of mature peripheral T-cells^6^.

HLA-DR is an antigen, part of class II MHC, which molecules are abundantly expressed in dendritic cells, monocytes and macrophages, and are responsible for antigen presentation to CD4^+^ T lymphocyte. Irritants are able to induce HLA-DR internalization by antigen-presenting cells^8^. There are dendritic cells located just beneath the epithelia, which express high levels of class II MHC molecules and are able to capture and respond against exogenous antigens. In response to these antigens, dendritic cells express chemokine receptor levels similar to the naive T cells. Therefore, dendritic cells are recruited into the T-cell zones of lymphoid tissues, the ideal site for antigen presentation to the recirculating T cells^6^. Inside the granulomas, macrophages are presented in two forms: some express lysozymes with a capacity for phagocytosis, while others represent antigen-presenting cells and CD4^+^ T cell-stimulating^6^. Amalgam tattoo attracts to its location many cells that express class II MHC molecules, enhancing HLA-DR molecule expression, which has previously been cited in this article.

While the presence of mucosa-associated lymphoid tissue (MALT) in the human upper respiratory mucosa is still being debated, it is known that lymphoid follicles are present in the mucosa of patients with chronic sinusitis, and they play an important role in mucosal immunity and persistent local inflammation^14^. Some studies have shown an increase of the HLA-DR expression in the nasal mucosa of patients with chronic sinusitis^7^, and an increase in CD4^+^ T lymphocytes expression^3,9^, thus contributing to the maintenance of the inflammatory process.

We believe that dendritic cells in the oral mucosa sensitized to amalgam tattoo compounds and with an increased HLA-DR expression migrate into lymphoid follicles present in the nasal mucosa of patients predisposed to chronic sinusitis. In this area, dendritic cells effectively stimulate the activation of CD4^+^ T lymphocytes, and, associated with new stimuli (infections, environmental conditions), leads to a profuse chronic inflammatory process causing a sinusitis crisis. Therefore, the removal of amalgam tattoo reduces the number of dendritic cells sensitized in these lymphoid follicles, since the migration from the oral cavity to these follicles ceases, minimizing or ending such sinusitis attacks, as occurred in the present case and in the case described by Weaver, et al.^16^ (1987).

## CONCLUSION

The amalgam tattoo seems to have a crucial relationship with chronic sinusitis in predisposed individuals. However, due to the rare cases described in the literature, the definitive establishment of this relationship can only be made by investigating the clinical profile in a large number of patients with amalgam tattoo.
